# Comparative effectiveness of nitinol staple-only fixation versus antiglide plate fixation for Weber type B distal fibular fractures

**DOI:** 10.1186/s12891-025-08835-1

**Published:** 2025-06-07

**Authors:** Kensei Yoshimoto, Masahiko Noguchi, Takumi Koseki, Ayako Tominaga, Ken Okazaki

**Affiliations:** 1 Orthopaedic Foot and Ankle Center, Shiseikai Daini Hospital, 5-19-1 Kamisoshigaya, Setagaya-ku, Tokyo, 157-8550 Japan; 2 Department of Orthopedic Surgery, Tokyo Women’s Medical University, 8- 1 Kawadacho, Shinjuku-ku, Tokyo, 162-0054 Japan

**Keywords:** Fibula fracture, Ankle fracture, Fracture fixation, Nitinol staples, Antiglide plates

## Abstract

**Background:**

Interest in less invasive surgeries for Weber type B distal fibular fracture has increased recently. This study aimed to demonstrate that nitinol staple-only fixation is less invasive compared to antiglide plate fixation.

**Materials and methods:**

This retrospective review involved 59 patients with Weber type B fibular fractures who underwent surgery between 2018 and 2023. Twenty-eight patients underwent antiglide plate fixation, whereas 31 underwent multiple nitinol staple-only fixation. The intraoperative assessment included skin incision length and operative time. The radiographic outcomes were bone union and fibular length. The clinical outcomes included delayed wound healing, infection, discomfort from the implant, implant removal, and the Self-Administered Foot Evaluation Questionnaire (SAFE-Q) score administered at the last visit.

**Results:**

The mean skin incision length and operative time of nitinol staple-only fixation were 3.8 ± 0.5 cm and 19.6 ± 3.6 min, compared with 8.7 ± 1.3 cm and 48.8 ± 10.6 min for plate fixation, respectively. Bone union was confirmed in all patients without fibular shortening. Although no significant differences in patients with delayed wound healing, infection, or postoperative SAFE-Q scores were found between the two groups, more patients with plate fixation reported discomfort from the implant (71.4% vs. 32.3%) and requested its removal (75.0% vs. 35.5%).

**Conclusion:**

Multiple nitinol staple-only fixations offer the advantages of a smaller skin incision, shorter operative time, lesser discomfort from the implants, and a reduced need for implant removal compared with antiglide plate fixation. Furthermore, staple-only fixation could achieve bone union without loss of correction. This suggests that multiple nitinol staple-only fixation may be less invasive and more beneficial for patients than antiglide plate fixation.

## Introduction

Ankle fractures are one of the most common injuries [1]. In patients with unstable ankle fractures, anatomical reduction of fibular fractures is important for achieving good functional recovery and preventing the development of osteoarthritis [2].

Fibular fractures can be classified according to the Danis–Weber classification, which describes the fracture pattern relative to syndesmosis. Weber type A, B, and C fractures are fractures located below the syndesmosis, at the level of the syndesmosis, and above the syndesmosis, respectively [3]. Weber type B fibular fractures are the most common type, accounting for up to 42% of all ankle fractures [4]. Historically, internal fixation of Weber type B fibular fractures has been achieved via plate fixation [5, 6]. However, plate fixation is invasive because of the long skin incision and increases the risk of wound healing complications and infections, with reported incidence rates of 16% [7–9]. Moreover, plate fixation requires more implant removal than screw-only fixation does because of discomfort [10]. Therefore, less invasive surgeries, including screw-only fixation or use of intramedullary nail, for Weber type B fibular fractures have gained much interest.

Recently, multiple nitinol staple fixation has been reported as a simple and effective approach for fracture fixation and foot/ankle surgery [11–13], and it can provide a high compression force and consistent bending stiffness, as seen in biomechanical studies using polyurethane foam [14, 15]. Therefore, nitinol staples were expected to be effective for Weber type B fibular fractures. However, no clinical studies have reported the effectiveness of nitinol staples for Weber type B fibular fractures.

This study aimed to assess the effectiveness of multiple nitinol staple-only fixations for Weber type B fibular fractures compared with antiglide plate fixation. Antiglide plate fixation might be more appropriate to compare than lateral neutralization plate fixation because it offers advantages for patients without osteoporosis, including less discomfort from the implant, fewer reoperations, and higher torque to failure [6]. We hypothesized that multiple nitinol staple-only fixation required shorter skin-incision length and operative time, and could achieve bone union without loss of correction.

## Materials and methods

### Subjects

A total of 84 patients with Weber type B distal fibular fractures who underwent surgery by eight surgeons (four senior and junior surgeons each) between 2018 and 2023, comprising 42 patients with antiglide plates and 42 with multiple nitinol staples, were included. Surgery was considered in cases of unstable fractures, fibular shortening or rotational displacement, and when patients preferred an early recovery. Patients with comminuted fractures (fractures in more than three parts) were not included because these fractures were fixed with an angular stable locking plate.

Patients with pilon fractures (2 ankles), syndesmotic injury which was diagnosed using the external rotation stress test under intraoperative fluoroscopy following internal fixation of fractures (3 ankles), fixation for posterior malleolus fractures which was performed to stabilize syndesmosis (5 ankles), and inadequate (< 1 year) postoperative follow-up (15 ankles) were excluded. Ultimately, 28 patients with plate fixation and 31 patients with multiple nitinol staple-only fixations were included in this study.

### Procedure

Internal fixation was performed using antiglide A.L.P.S. distal fibular composite plate (Zimmer Biomet, Warsaw, IN, USA) or locking third tubular plate (Arthrex, Naples, FL, USA) (from 2018 to 2020 and multiple DynaNite nitinol staples (Arthrex, Naples, FL, USA) only from 2021 to 2023. We considered that contraindication of the nitinol staple-only fixation was comminuted fractures (fractures in more than three parts). Only one patient underwent surgery using an antiglide distal fibular plate between 2021 and 2023 because the bone was initially cracked during the repositioning of the fracture.

**Fig. 1:**
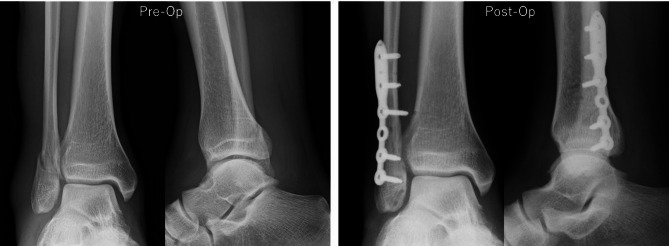
Preoperative and postoperative anteroposterior and lateral radiographs of a patient who underwent antiglide plate fixation

**Fig. 2:**
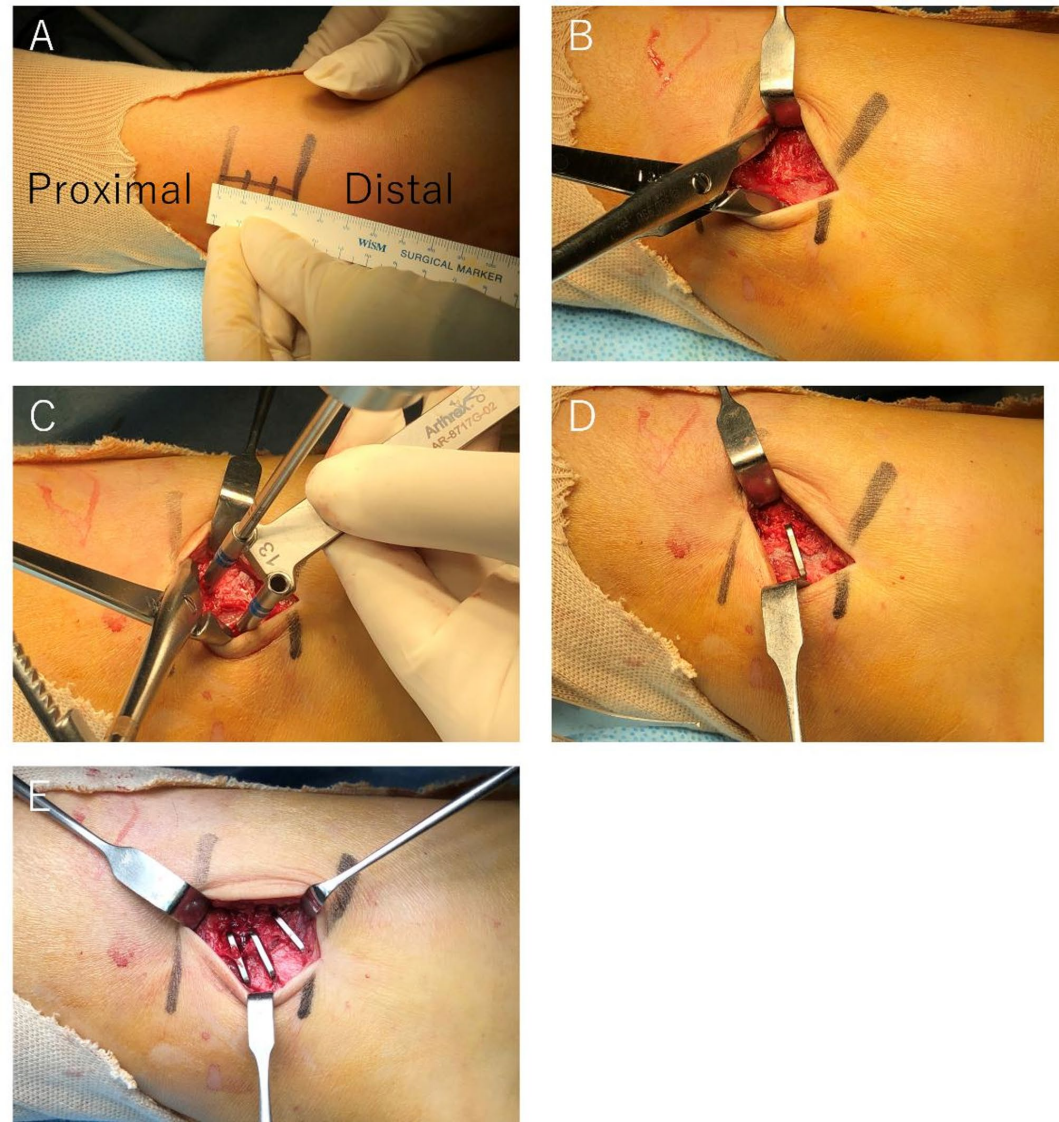
Fixation technique using nitinol staples. The initial 3-cm skin incision (**A**). The fracture was reduced using a bone clamp forceps (**B**) and fixed by placing the nitinol staple at the center, perpendicular to the fracture line after drilling (**C** and **D**). The bone clamp forceps were removed after fixation with a single staple, and two additional staples were placed in posterior and anterior fracture sites (**E**)

**Fig. 3:**
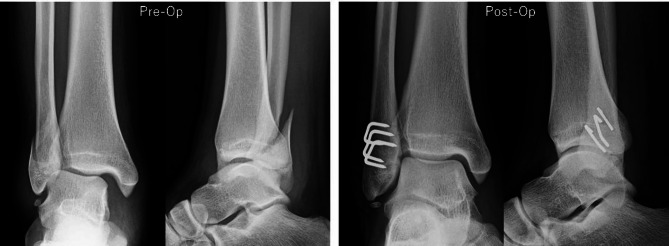
Preoperative and postoperative anteroposterior and lateral radiographs of a patient who underwent fixation with multiple nitinol staples

All procedures were performed under general anesthesia. Antiglide plate fixation was performed in supine position using a posterolateral approach. A distal fibular composite plate was typically applied with three or four bicortical screws proximally and at least two locking screws distally (Fig. 1). Multiple nitinol staple-only fixations were performed in spine position using a lateral approach with a skin incision between the proximal and distal ends of the fracture. If a patient has a long oblique fracture, we initially performed a 3-cm skin incision and extended it as needed (Fig. 2A). The fracture was reduced using a bone clamp forceps (Fig. 2B) and fixed by placing the nitinol staple at the center, perpendicular to the fracture line after drilling (Fig. 2C and D). The bone clamp forceps were removed after fixation with a single staple, and then, two additional staples were placed in posterior and anterior fracture sites (Figs. 2E and 3). The size of the nitinol staples used in this cohort was either 13 mm × 10–15 mm × 12 mm. Standard closure was carried out in all surgeries. All patients with medial malleolar fractures were reduces and fixed with 4-mm cancellous screws or Kirschner wires.

Postoperatively, patients were allowed weight bearing as tolerated from day 1 after surgery with an ankle brace [16, 17]. The ankle brace was removed 12 weeks after the surgery. All patients consulted a physical therapist postoperatively for exercise therapy and received advice on how to start mobilizing according to their specific postoperative care regimen.

### Outcome measures

The intraoperative assessment findings included the skin incision length for fibular fractures and operative time from skin incision to closure for fibular fractures.

**Fig. 4:**
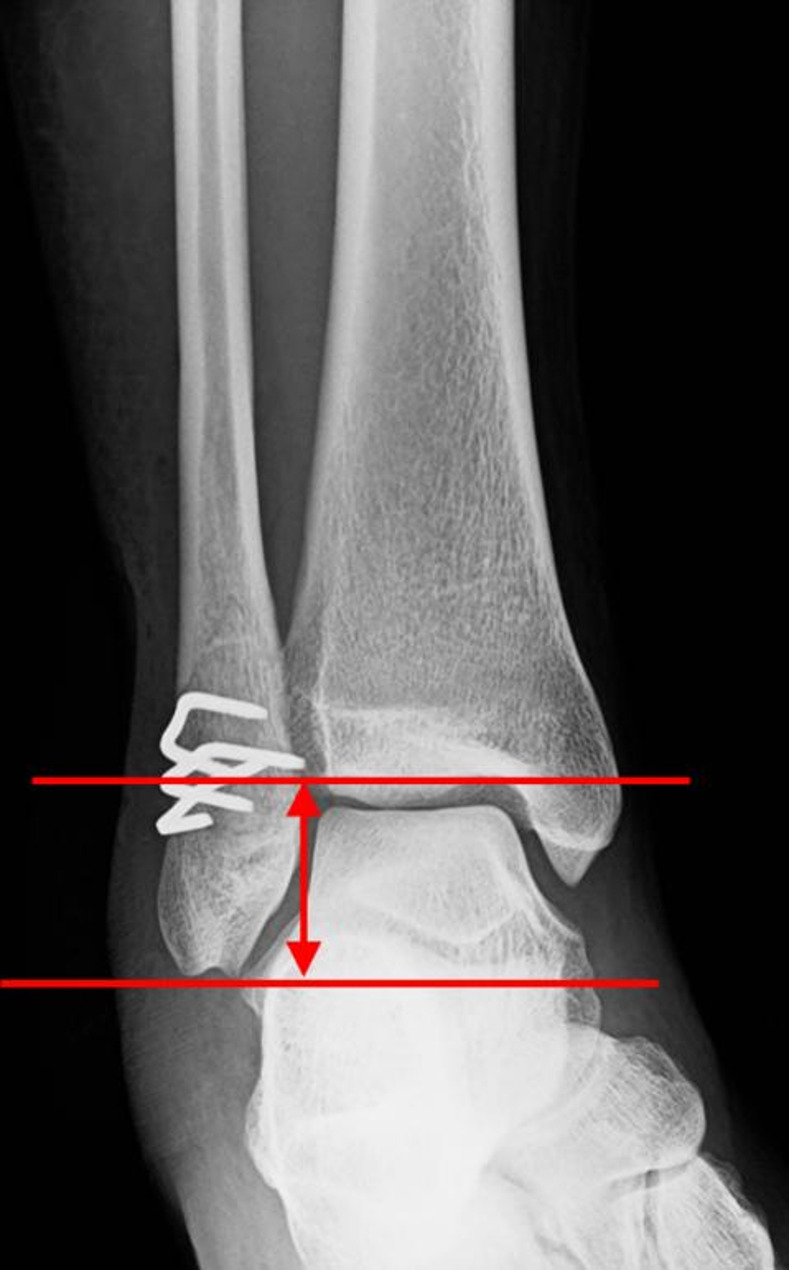
The fibular length is defined as the distance from the fibular tip to the tibial articular surface

Radiographic outcomes were bone union and fibular length. If there was no evidence of implant loosening, the presence of a fracture line, or bone resorption on plain anteroposterior and lateral ankle radiographs six months after surgery, it was concluded that bone union had been achieved. The fibular length, which is the distance from the fibular tip to the tibial articular surface (Fig. 4) [17], was measured using a picture archiving and communication system to evaluate fibular shortening immediately after surgery and at the last visit on standard anteroposterior ankle radiographs.

The clinical outcomes included delayed wound healing, infection, discomfort from the implant, implant removal, and Self-Administered Foot Evaluation Questionnaire (SAFE-Q) score [18, 19]. The SAFE-Q [18, 19] was used for the clinical assessment at the last visit. SAFE-Q contains 34 questions on foot and ankle symptoms, which are divided into the following five subscales: pain and pain-related; physical functioning and daily living; social functioning; shoe-related; and general health and well-being. Each subscale score ranges from 0 to 100 points [19]. Radiographic assessment was performed immediately after surgery and at the last visit using standard anteroposterior ankle radiographs. The fibular length, which is the distance from the fibular tip to the tibial articular surface (Fig. 4) [20], was measured to evaluate fibular shortening. Bone union was confirmed on standard anteroposterior and lateral ankle radiographs at the last visit.

### Statistical analysis

Statistical analysis was performed using JMP 14.0 software (SAS Institute, Cary, NC, USA). The data obtained by comparing the categorical and continuous variables between the antiglide plating and nitinol staple groups were examined using Fisher’s exact and Mann–Whitney U tests, respectively. *P* values < 0.05 were accepted as statistically significant.

The intraclass correlation coefficients (ICC) and their 95% confidence intervals (CIs) were used for the intra- and interobserver reproducibility of the fibular length. Two orthopedic surgeons measured fibular length, and one surgeon measured two times at intervals of 1 month. The interpretation of ICC values was as follows: 1.0, perfect agreement; 0.81–0.99, excellent agreement; 0.61–0.80, good agreement; 0.41–0.60, moderate agreement; 0.21–0.40, fair agreement; 0.00–0.20, poor agreement [21]. The paired t-test was used to analyze the comparison of the fibular length immediately after surgery and at the latest visit.

A post hoc power analysis, which compared the skin incision length and operative time between patients undergoing plate fixation and those receiving multiple nitinol staple-only fixation, demonstrated a statistical power exceeding 0.8.

## Results

Among 59 patients, 28 were female and 31 were male, with an average age of 45.9 years (range, 15–88) and an average body mass index of 23.3 kg/m^2^ (range, 15.6–29.7) at the time of operation. The mean follow-up period was 13.4 months, with a range of 12–24 months. There were no obese patients (body mass index > 30 kg/m²), but there were 13 patients who smoked and 5 patients with diabetes. No significant differences in the baseline characteristics of the patients were found between the antiglide plate fixation and multiple nitinol staple-only fixation groups (Table 1).

The mean skin incision length and operative time of the nitinol staple-only fixation group were 3.8 cm and 19.6 min, respectively, which were significantly smaller and shorter than those of the plate fixation group with 8.7 cm and 48.8 min, respectively.

All patients in both groups achieved bone union. The intra- and interobserver ICCs for fibular length were 0.93 (95% CI, 0.79–0.98) and 0.95 (95% CI, 0.86–0.98), respectively. The 95% CIs of the differences in fibular lengths obtained immediately after surgery from those obtained at the last visit were − 0.16–0.38 mm in the antiglide plating group and − 0.10–0.15 mm in the nitinol staple only group. These values were similar to the 95% CI values of the intra- and interobserver measurement errors (− 0.22–0.62 mm and − 0.43–0.27 mm) (Table 2).

Although no significant differences in the number of patients with delayed wound healing and infection were noted, more patients with plate fixation felt uncomfortable with the implant and requested its removal. All patients in both groups achieved bone union, and no significant differences in postoperative SAFE-Q scores were found between the two groups (Table 3).

**Table 1:** Comparison of the patients’ demographic characteristics between the plate fixation and nitinol staple fixation groups

	Plate (*n* = 28)	Staple (*n* = 31)	*P* value
Age	47.8 ± 19.9 (15 to 83)	44.3 ± 18.6 (16 to 88)	0.3825
Sex(female/male)	13/15	15/16	1
BMI (kg/m^2^)	23.3 ± 3.0 (15.6 to 28.7)	23.2 ± 3.0 (18.9 to 29.7)	0.6271
Follow-up duration (months)	13.6 ± 3.5 (12 to 24)	13.2 ± 3.2 (12 to 24)	0.2218
Medial malleolus fracture	3/28	4/31	1
Posterior malleolus fracture	5/28	8/31	0.5398
Obesity (BMI > 30 kg/m²)	0/28	0/31	1
Smoking	6/28	7/31	1
Diabetes	2/28	3/31	1

Values are expressed as mean ± standard deviation (minimum to maximum)

*P <.05

**Table 2:** Fibular length obtained immediately after surgery and at the last visit

	Immediately after surgery	Last visit	95% CI of differences
Plate	28.0 ± 2.7 (95% CI, 27.0 to 29.1)	28.1 ± 2.6 (95% CI, 27.1 to 29.1)	-0.16 to 0.38
Staple	28.2 ± 2.5 (95% CI, 27.3 to 29.2)	28.0 ± 2.1 (95% CI, 27.2 to 28.8)	-0.10 to 0.15

CI: confidential interval

Values are expressed mean ± standard deviation

**Table 3:** Comparison of the intra- and postoperative outcomes between the patients with plate fixation and those with nitinol staple fixation

	Plate (*n* = 28)	Staple (*n* = 31)	*P* value
Operative time for fibular fracture (minutes)	48.8 ± 10.6 (30 to 70)	19.6 ± 3.6 (15 to 29)	< 0.0001*
Skin incision length for fibular fracture (cm)	8.7 ± 1.3 (7.0 to 10.2)	3.8 ± 0.5 (3.0 to 5.0)	< 0.0001*
Bone union	28/28 (100%)	31/31 (100%)	1
Delayed wound healing	8/28 (28.6%)	3/31 (9.7%)	0.0949
Infection	2/28 (7.1%)	1/31 (3.2%)	0.5995
Discomfort from implant	20/28 (71.4%)	10/31 (32.3%)	0.0040*
Implant removal	21/28 (75.0%)	11/31 (35.5%)	0.0038*
SAFE-Q			
Pain	90.8 ± 15.0 (31.1 to 100)	93.6 ± 12.2 (54.4 to 100)	0.3505
Physical functioning	94.8 ± 8.8 (56.8 to 100)	96.1 ± 7.8 (70.5 to 100)	0.2730
Social functioning	96.9 ± 8.2 (62.5 to 100)	96.4 ± 11.1 (45.8 to 100)	0.8575
Shoe-related	92.6 ± 13.2 (50.0 to 100)	93.6 ± 12.9 (58.3 to 100)	0.6194
General health	93.3 ± 13.2 (45.0 to 100)	94.3 ± 11.3 (55.0 to 100)	0.7353

SAFE-Q: Self-Administered Foot Evaluation Questionnaire

Values are expressed as mean ± standard deviation (minimum to maximum)

*P <.05

## Discussion

To the best of our knowledge, this study was the first to assess the effectiveness of multiple nitinol staple-only fixations for Weber type B distal fibular fractures. Our study revealed that staple-only fixation requires a shorter operative time and a smaller skin incision than traditional plate fixation and can achieve bone union without loss of correction. Furthermore, fewer patients with nitinol staple-only fixation complained of discomfort from the implant and requested its implant removal than those with plate fixation.

The plate fixation for Weber type B distal fibular fractures is a traditional and universal procedure [5, 6]. However, whether invasive plate fixation for Weber type B distal fibular fractures is optimal remains a concern. Recently, several studies have reported the effectiveness of less invasive fibular intramedullary nail fixation [22]. A cadaveric biomechanical study showed comparable biomechanical properties when testing the fibular intramedullary nail and distal fibular locking plate to failure [23]. Furthermore, fibular intramedullary nail fixation requires a shorter skin incision [16], could reduce the risk of wound complications, and could provide favorable clinical outcomes compared with plate fixations [22].

The effectiveness of less invasive screw-only fixation for Weber type B distal fibular fractures has been reported. A previous biomechanical study demonstrated that distal fibular fractures fixed with multiple lag screws performed comparably to those fixed with a neutralization plate [24]. A clinical study reported that screw-only fixation was equally effective as lateral plating in achieving fracture union without loss of reduction and provided lesser soft tissue dissection, less prominent, symptomatic, and palpable hardware, and a reduced requirement for secondary surgical removal [8, 25].

In the present study, delayed wound healing and infection occurred more frequently in the antiglide plate fixation group, but there were no significant differences. Although the effectiveness of nitinol staples for Weber type B fibular fractures is unclear, recent biomechanical studies using polyurethane foam have revealed that nitinol staple fixation could provide more compression force at the fracture site than screw-only and plate fixations [14, 15], and that double-staple fixation could provide the most consistent bending stiffness in all planes compared to single-staple or plate fixation [14]. Another cadaveric study revealed that multiple staple fixation can provide biomechanical performance comparable to that of established crossed-screw and plate-and-screw fixation techniques used for fusion of the first tarsometatarsal joint [26]. Furthermore, a recent systematic review showed that nitinol staple fixation in foot surgery can achieve an acceptable fusion rate [13]. While these studies did not employ a distal fibular fracture model, these results suggest that multiple nitinol staples could provide adequate strong fixation. This study supports the findings of these biomechanical studies demonstrating the efficacy of multiple nitinol staple-only fixations for achieving bone union without loss of correction in patients with Weber type B fibular fractures.

The present study also found that, compared with plate fixation, internal fixation with nitinol staples had the advantages of a smaller skin incision and shorter operative time. The mean skin incision length of nitinol staple-only fixation was 3.8 cm, which was relatively shorter than that of the screw-only fixation that was reported to be 6.2 cm [25]. Furthermore, the mean operative time of nitinol staple-only fixation was 19.6 min, which was also relatively shorter than those obtained for screw-only and intramedullary nail fixations, reported to be 69.6 and 49 min, respectively [25, 27].

In the present study, fewer patients with nitinol staple-only fixation complained of discomfort from the implant and requested its removal compared with those with plate fixation. However, the odds of complaining of discomforts and requesting implant removal in the patients with plate fixation was relatively higher than those reported in previous studies [6, 8]. In Japan, medical costs borne by patients were low because of the national health insurance system and the high-cost medical expense benefit, which cover all Japanese citizens. Therefore, patients easily complain of trivial discomfort from the implant and request implant removal.

This study had several limitations. First, it retrospectively analyzed a small case series. However, the power analysis revealed sufficient statistical power. Second, differences in surgeon skills and expertise may introduce bias. Third, only short-term outcomes were recorded. Long-term follow-up could influence several outcomes. However, the short-term follow-up did not influence the primary outcomes, including skin incision length and operative time, of this study. Fourth, nitinol staple-only fixation was not applicable to comminuted fractures, which could have caused selection bias in our study. However, because patients with comminuted fractures were not included in this study, and only one patient could not undergo nitinol staple-only fixation due to a bone crack during the repositioning of the fracture, we believed that this selection bias can be ignored. Fifth, bone union was confirmed on plain radiographs; however, computed tomography was required for accurate confirmation of bone union. Sixth, osteoporosis wasn’t evaluated in this study, and currently, there’s no supporting evidence for using staple-only fixation in osteoporotic bone. Surgeons should be careful when choosing this method for such patients. Finally, the fibular length on the unaffected side was ultimately not assessed. Comparing both sides would have helped ensure the fracture reduction was done properly.

## Conclusions

In conclusion, this study revealed that multiple nitinol staple-only fixations for Weber type B distal fibular fractures offers smaller skin incision, shorter operative time, lesser discomfort from implants, and a reduced need for implant removal compared with antiglide plate fixation. Furthermore, staple-only fixation could achieve bone union without loss of correction. Thus, multiple nitinol staple-only fixations may be less invasive and more beneficial for patients than antiglide plate fixation.

## Data Availability

The data that support the findings of this study are available from the corresponding author, KY, upon reasonable request.

## References

[1] Court-Brown CM, Caesar B. Epidemiology of adult fractures: a review. Injury. 2006;37:691–7. 16814787 10.1016/j.injury.2006.04.130

[2] Gu S, Wang S, Gong Y, Ren Y, Feng H. Numerical simulations of the effect of lateral malleolus fracture malunion on ankle biomechanics: different offset directions and offsets. Foot Ankle Surg. 2024;30:135–44. 37919180 10.1016/j.fas.2023.10.007

[3] Hughes JL, Weber H, Willenegger HKE. Evaluation of ankle fractures: non-operative and operative treatment. Clin Orthop Relat Res. 1979;138:11–9. 445892

[4] Han SM, Wu TH, Wen JX, Wang Y, Cao L, Wu WJ, et al. Radiographic analysis of adult ankle fractures using combined Danis-Weber and Lauge-Hansen classification systems. Sci Rep. 2020;10:7655. 32376947 10.1038/s41598-020-64479-2 PMC7203210

[5] Giver Jensen T, Aqeel Khudhair Almadareb M, Booth Nielsen M, Jesper Hansen E, Lindberg-Larsen M. Outcome after treatment of distal fibula fractures using one-third tubular plate, locking compression plate or distal anatomical locking compression plate. J Foot Ankle Surg. 2023;62:524–8. 36642663 10.1053/j.jfas.2022.12.008

[6] Deng Y, Staniforth TL, Zafar MS, Lau YJ. Posterior antiglide plating vs lateral neutralization plating for Weber B distal fibular fractures: a systematic review and meta-analysis of clinical and Biomechanical studies. Foot Ankle Int. 2022;43:850–9. 35373597 10.1177/10711007221079617

[7] Fenelon C, Galbraith JG, Fahey T, Kearns SR. The operative treatment of ankle fractures: a 10-year retrospective study of 1529 patients. J Foot Ankle Surg. 2021;60:663–8. 33509713 10.1053/j.jfas.2020.03.026

[8] McKenna PB, O’Shea K, Burke T. Less is more: lag screw only fixation of lateral malleolar fractures. Int Orthop. 2007;31:497–502. 16947052 10.1007/s00264-006-0216-6 PMC2267624

[9] White TO, Bugler KE, Appleton P, Will E, McQueen MM, Court-Brown CM. A prospective randomised controlled trial of the fibular nail versus standard open reduction and internal fixation for fixation of ankle fractures in elderly patients. Bone Jt J. 2016;98–B:1248–52. 10.1302/0301-620X.98B9.35837 27587528

[10] Lindsjö U, Tornetta P, Creevy WR. Lag screw only fixation of the lateral malleolus. J Orthop Trauma. 2001;15:593–4. 11770499 10.1097/00005131-200111000-00018

[11] Wu JC, Mills A, Grant KD, Wiater PJ. Fracture fixation using shape-memory (ninitol) staples. Orthop Clin North Am. 2019;50:367–74. 31084839 10.1016/j.ocl.2019.02.002

[12] Schipper ON, Ellington JK. Nitinol compression staples in foot and ankle surgery. Orthop Clin North Am. 2019;50:391–9. 31084842 10.1016/j.ocl.2019.02.003

[13] Reddy AR, Hampton H, Dzieza WK, Toussaint RJ. Nitinol compression staples in foot orthopaedic surgery: a systematic review. Foot Ankle Orthop. 2024;9:24730114241300160. 39619115 10.1177/24730114241300158 PMC11607750

[14] Hoon QCJ, Pelletier MH, Christou C, Johnson KA, Walsh WR. Biomechanical evaluation of shape-memory alloy staples for internal fixation—an in vitro study. J Exp Orthop. 2016;3:19. 27578288 10.1186/s40634-016-0055-3 PMC5005248

[15] Aiyer A, Russell NA, Pelletier MH, Walsh WR, NA AA. The impact of nitinol staples on the compressive forces, contact area, and mechanical properties in comparison to a claw plate and crossed screws for the first tarsometatarsal arthrodesis. Foot Ankle Spec. 2016;9:232–40. 26655080 10.1177/1938640015620655

[16] Smeeing DPJ, Houwert RM, Briet JP, Groenwold RHH, Lansink KWW, Leenen LPH, et al. Weight-bearing or non-weight-bearing after surgical treatment of ankle fractures: a multicenter randomized controlled trial. Eur J Trauma Emerg Surg. 2020;46:121–30. 30251154 10.1007/s00068-018-1016-6 PMC7026225

[17] Le V, Viskontas D, Lohre R, Yan J, Stone T, Perey B, et al. Immediate unprotected weightbearing vs 2 weeks nonweightbearing after open reduction internal fixation of ankle fractures. Foot Ankle Int. 2024;45:103–14. 38156640 10.1177/10711007231217675

[18] Niki H, Haraguchi N, Aoki T, Ikezawa H, Ouchi K, Okuda R, et al. Responsiveness of the self-administered foot evaluation questionnaire (SAFE-Q) in patients with hallux valgus. J Orthop Sci. 2017;22:737–42. 28501433 10.1016/j.jos.2017.04.005

[19] Niki H, Tatsunami S, Haraguchi N, Aoki T, Okuda R, Suda Y, et al. Validity and reliability of a self-administered foot evaluation questionnaire (SAFE-Q). J Orthop Sci. 2013;18:298–320. 23299996 10.1007/s00776-012-0337-2 PMC3607735

[20] Chiang CC, Tzeng YH, Jeff Lin CF, Wang CS, Lin CC, Chang MC. Arthroscopic reduction and minimally invasive surgery in supination–external rotation ankle fractures: a comparative study with open reduction. Arthroscopy. 2019;35:2671–83. 31500754 10.1016/j.arthro.2019.03.051

[21] Yoshimoto K, Noguchi M, Maruki H, Tominaga A, Okazaki K. Hindfoot alignment and ankle stability following arthroscopic lateral ankle ligament repair. Foot Ankle Int. 2023;44:872–8. 37391997 10.1177/10711007231181123

[22] Asloum Y, Bedin B, Roger T, Charissoux JL, Arnaud JP, Mabit C. Internal fixation of the fibula in ankle fractures. A prospective, randomized and comparative study: plating versus nailing. Orthop Traumatol Surg Res. 2014;100:S255–9. 24709304 10.1016/j.otsr.2014.03.005

[23] Carter TH, Wallace R, Mackenzie SA, Oliver WM, Duckworth AD, White TO. The fibular intramedullary nail versus locking plate and lag screw fixation in the management of unstable elderly ankle fractures: a cadaveric Biomechanical comparison. J Orthop Trauma. 2020;34:e401–6. 33065664 10.1097/BOT.0000000000001814

[24] Misaghi A, Doan J, Bastrom T, Pennock AT. Biomechanical evaluation of plate versus lag screw only fixation of distal fibula fractures. J Foot Ankle Surg. 2015;54:896–9. 25990534 10.1053/j.jfas.2015.03.011

[25] Paez CJ, Lurie BM, Bomar JD, Upasani VV, Pennock AT. Plate versus lag screw only fixation of unstable ankle fractures involving the fibula in adolescent patients. J Pediatr Orthop. 2021;41:e161–6. 33165263 10.1097/BPO.0000000000001702

[26] Sands A, Zderic I, Swords M, Gehweiler D, Ciric D, Roth C, et al. First tarsometatarsal joint fusion in foot—a Biomechanical human anatomical specimen analysis with use of low-profile nitinol staples acting as continuous compression implants. Med. 2023;59:1310. 10.3390/medicina59071310 PMC10383077 37512121

[27] Bäcker HC, Vosseller JT. Fibula fracture: plate versus nail fixation. Clin Orthop Surg. 2020;12:529–34. 33274031 10.4055/cios19177 PMC7683182

